# Effects of curcumin on glucose metabolism in the brains of rats subjected to chronic unpredictable stress: a ^18^ F-FDG micro-PET study

**DOI:** 10.1186/1472-6882-13-202

**Published:** 2013-08-01

**Authors:** Zheng Lin, Ligen Shi, Jing Lu, Jinhui Li, Hua Hu, Chuantao Zuo, Weijun Tang, Yunrong Lu, Aimin Bao, Lei Xu

**Affiliations:** 1 Department of Psychiatric, Second Affiliated Hospital of Zhejiang University School of Medicine, Hangzhou, Zhejiang, China; 2 Key Laboratory of Medical Neurobiology of Ministry of Health of China, Zhejiang University School of Medicine, Hangzhou, Zhejiang, China; 3 Department of Chinese Medicine & Rehabilitition, Second Affiliated Hospital of Zhejiang University School of Medicine, Hangzhou, Zhejiang, China; 4 Department of Neurology, The Second Affiliated Hospital, Soochow University, Suzhou city, Jiangsu Province, China; 5 Pet Center, Department of Nuclear Medicine, Huashan Hospital, Fudan University, Shanghai, China; 6 Department of Radiology, Huashan hospital, Fudan University, Shanghai, China; 7 Department of Geriatric Diseases, Second Affiliated Hospital of Zhejiang University School of Medicine, Hangzhou, Zhejiang 310009, China

## Abstract

**Background:**

Chronic unpredictable stress (CUS) can cause behavioral and physiological abnormalities that are important to the prediction of symptoms of depression that may be associated with cerebral glucose metabolic abnormalities. Curcumin showed potential antidepressant effects, but whether or not it can reverse cerebral functional abnormalities and so ameliorate depression remains unknown.

**Methods:**

To investigate the effects of curcumin on brain activity in CUS rats, rats were subjected to 3 weeks of CUS and then treated with curcumin orally at a dose of 40 mg/kg/day for one month. ^18^ F fluorodeoxyglucose (^18^ F-FDG)-micro positron emission tomography (micro-PET) neuroimaging was used to detect changes in cerebral metabolism. Body weight, sucrose preference, and open field tests were used to record depressive behaviors during CUS and after curcumin treatment.

**Results:**

Three weeks of CUS significantly decreased body weight, sucrose preference, sucrose consumption, total distance travelling, and the number of rearing events. It also induced metabolic alterations in several parts of the brain, showing increased glucose metabolism in the right hemisphere. After curcumin treatment for one month, sucrose preference, sucrose consumption, total distance travelling, and the number of rearing events returned to normal levels. Curcumin treatment also induced strong deactivation of the left primary auditory cortex and activation of amygdalohippocampal cortex.

**Conclusion:**

Curcumin was found to ameliorate the abnormalities in the behavior and brain glucose metabolism caused by CUS, which may account for its antidepressive effects.

## Background

Depression is one of the most burdensome diseases in the world [1]. It has been recognized as the alternation of monoamine neurotransmitters serotonin and norepinephrine in the brain [2]. Antidepressants that regulate the monoamine neurotransmitter system have been used successfully in clinical settings, but 20–30% of depressed patients do not experience a satisfactory level of amelioration [3]. For this reason, searching for an alternative biological antidepressant that would be safer and more effective is important. With the development of neuroimaging, many studies have depicted the limbic system in depression, showing changes in the structure and function of the prefrontal cortex, hippocampus, and amygdala [4]. These neuropathologic alterations may mediate or respond to the emotional, behavioral, and cognitive manifestations of depressive episodes. For this reason, there are reports of some antidepressants that ameliorate depression by reversing cerebral functional abnormalities [5].

Curcumin, a yellow plant pigment extracted from *Curcuma longa* (turmeric) rhizomes, has wide-ranging neuroprotective effects in the treatment of neuropsychiatric disorders, including Parkinson’s disease, Alzheimer’s disease, schizophrenia, and epilepsy [6]. It is been reported that curcumin can penetrate the blood–brain barrier and modulate the levels of neurotransmitters like norepinephrine, dopamine, and serotonin in the brain [3]. The antidepressive effects of curcumin have been demonstrated in a variety of animal models, such as the forced swimming test [7,8], tail suspension [9], olfactory bulbectomy model [10], and chronic unpredicted stress (CUS) [11]. The mechanisms by which curcumin counteracts depression might be associated with several signaling pathways. For example, it is probable that a monoamine oxidase inhibitor modulates the serotonin and dopamine levels [7,8], blocks the release of glutamate from rat prefrontal cortex nerve terminals [12], increases the concentrations of brain-derived neurotrophic factor in the hippocampus and prefrontal cortex, and promote neurogenesis [13]. Curcumin is also believed to have an effect on diabetes mellitus by inhibiting glucose uptake [14]. However, there is little information regarding whether curcumin can reverse dysfunction of the cerebral glucose metabolism, which was impaired in depression.

^18^ F fluorodeoxyglucose (^18^ F-FDG) is a high spatial resolution tracer which can be used to perform animal micro-PET imaging, which was used to investigate rat brain activity during acute and chronic stress [15,16]. In the present study, micro-PET imaging was performed to investigate changes in behavior and brain glucose metabolism during depression after treatment with curcumin in a CUS rat model, which is widely used to evaluate the effects of antidepressants in vivo [17]. In the present study, we want to use ^18^ F-FDG micro-PET imaging to further explore the effects of curcumin on the behavioral and brain glucose metabolism caused by CUS, which will help to know the anti-depressive mechanism of curcumin and promote its translational development.

## Methods

### Animal model

All experiments were performed in accordance with the National Institutes of Health Guide for the Care and Use of Laboratory Animals and they were approved by the Committee on Animal Care and Usage of Zhejiang University. A total of 27 male Sprague–Dawley rats with body weights ranging from 280 to 300 g were obtained from Shanghai Institutes for Biological Sciences. The rats were housed in a humidity- and temperature-controlled environment with 12-hour light/dark cycles and free access to food and water. Prior to the experiments, the rats were placed in a new environment for 7 days to allow them to habituate to their housing conditions.

After one-week acclimation, a CUS model was established as described previously in rats [18]. Control animals remained in a comparable environment. Unpredictable stress included water deprivation, empty water bottles, continuous lighting, tilting of the cages, crowded housing, damp bedding, white noise, and strobe lighting. These stress events took place between 9:00 and 10:00 a.m. every day. The specific experimental protocol is as stated in Table 1 and all the experiments lasted for three weeks.

**Table 1:** Chronic unpredictable stress (CUS) schedule

	**Sunday**	**Monday**	**Tuesday**	**Wednesday**	**Thursday**	**Friday**	**Saturday**
Water Deprivation	10:00 →	11:00					
Empty Water Bottle		08:00 → 09:00					
Continuous Lighting	10:00 →	8:00			17:00 →	10:00	
Cage Tilt			11:00 → 17:00				
Paired Housing	→	8:00		10:00 →	10:00	10:00 →	→
Damp Bedding				17:00 →	10:00		
White Noise						10:00 → 13:00	
Strobe Light		11:00 → 16:00		13:00 → 15:00			

### Experimental design

**Figure 1:**
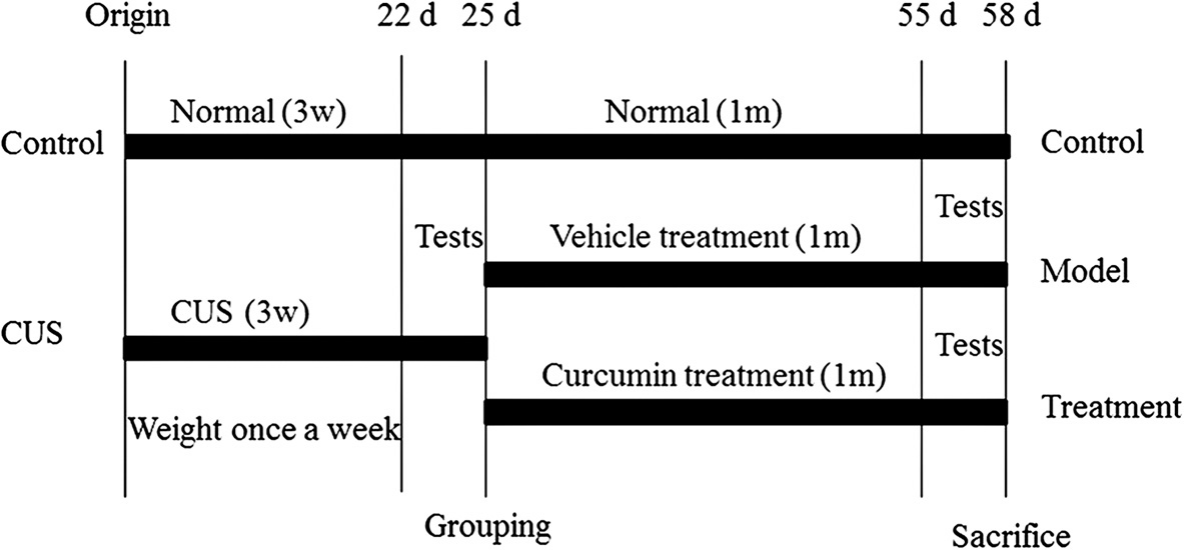
**Schedule of procedures used in the present study.** Tests include the open field test, sucrose preference test, and micro-PET.

Rats were randomly assigned into two groups: control (n = 14) and CUS (n = 13). After 3-week stress stimulation, CUS rats were further randomly divided into two groups: model (n = 6) and treatment (n = 7). Curcumin (purity ≥ 98%; Nanjing Zelang Medical Technological Co.Ltd., China), was dissolved in rice bran oil to make a 40 mg/kg solution [11,19]. Starting from the second month, the rats in the treatment group were given curcumin by oral gavage. The model group was fed rice bran oil. All experiments were performed in the morning from 9:00 to 11:00 a.m. The whole experimental procedure is shown in Figure 1.

### Open field testing

The open-field test protocol was performed as described in our earlier work [20]. Briefly, spontaneous activity was measured and recorded for 5 min in an open field to evaluate the difference between the groups. Briefly, the test was conducted on white painted plywood (72 cm × 72 cm) with walls 36 cm high in a soundproof room with dim lighting. The rats had not undergone previous habituation to this room. The base was divided into 16 squares using white strips. These constituted the peripheral and central sectors. Rats were initially placed in the central sector and then released to explore the facility for a 5 min period, during which the parameters (e.g. total distance travelling, the number of rearing events) were monitored using a VideoMot2–Video Activity Measuring System (TSE systems GmbH, Germany).

### Sucrose preference testing

The sucrose preference test procedure was performed as previously described [21]. The rats were initially acclimatized to sucrose and water for three days. For the test, rats were fed 5% sucrose solution (w/v) as follows: two bottles of 5% sucrose solution were placed in each cage for the first day; one bottle of water and one of sucrose were placed in each cage on the second day, and the positions of the bottles were switched to eliminate the effects of side preference. On the third day, rats were deprived of food and water for 24 h. On the fourth day, rats were given one bottle of water and one bottle of sucrose solution. Intakes of water and sucrose solution were quantified by measurement of bottle volumes before and after the test. After 24 h, the volumes of sucrose solution and water consumed were recorded, and the sucrose preference was calculated using the following formula: Sucrose preference = sucrose consumption/(water consumption + sucrose consumption) × 100%.

### Micro-PET scan

The micro-PET scan protocol was performed as described in the previous study [15]. Before ^18^ F-FDG injection, the rats were fasted for 8–12 h with free access to water in order to enhance the uptake of the tracer by the brain and to reduce the interference of other systems, like skeletal muscle [22]. Each rat was placed on a heating pad which was kept at 30°C for at least 30 min prior to ^18^ F-FDG injection. Rats were briefly anesthetized using halothane gas (1.5 min) in order to maximize tracer uptake by the brain. The rats were then injected with approximately 18.5 MBq (500 μCi) of ^18^ F-FDG through the tail vein either before administration of curcumin or 0, 2, or 4 weeks after CUS. After 40 min, the rats were placed in a spread-legged prone position with halothane gas anesthesia (5% induction and 1.5% for maintenance) of micro PET R4 (Kyowa micro Knoxville, TN, U.S.), which consists of a ring of 96 position-sensitive γ-ray scintillation detectors 15 cm in diameter, providing a 10.8 cm trans-axial, and a 7.8 cm axial field of view, with an intrinsic resolution < 1.8 mm. The entire static process continued for 15 min.

### Statistical analysis

Areas of the brain were manually extracted from all micro-PET images. These images were normalized using ^18^ F-FDG rat brain templates. In order to obtain accurate anatomical information, statistical parametric mapping (SPM5) software for PET template normalization into magnetic resonance imaging (MRI) template was used. This system was placed in stereotaxic space [23]. To increase statistical power, all normalized images were smoothed with an isotropic Gaussian kernel (2 mm full width at half-maximum). Voxel-based statistical analyses were carried out with the SPM5 software.

Because the data was not always normally distributed, the difference between the groups was statistically evaluated using nonparametric testing. Body weight was analyzed using a repeated measurement ANOVA (CUS and control groups served as independent factors and time served as a repeated measure). The effects of CUS on sucrose preference, sucrose consumption, total distance travelling, and the number of rearing events were analyzed using the Mann–Whitney U-test. All behavioral results were calculated using the median values. All tests were two-tailed and the *P* values less than 0.05 were considered significant. Statistical analyses were performed with SPSS version 18.0 (SPSS, Inc., Chicago, IL, U.S.).

## Results

### Effects of CUS and curcumin on body weight and behavior tests

**Figure 2:**
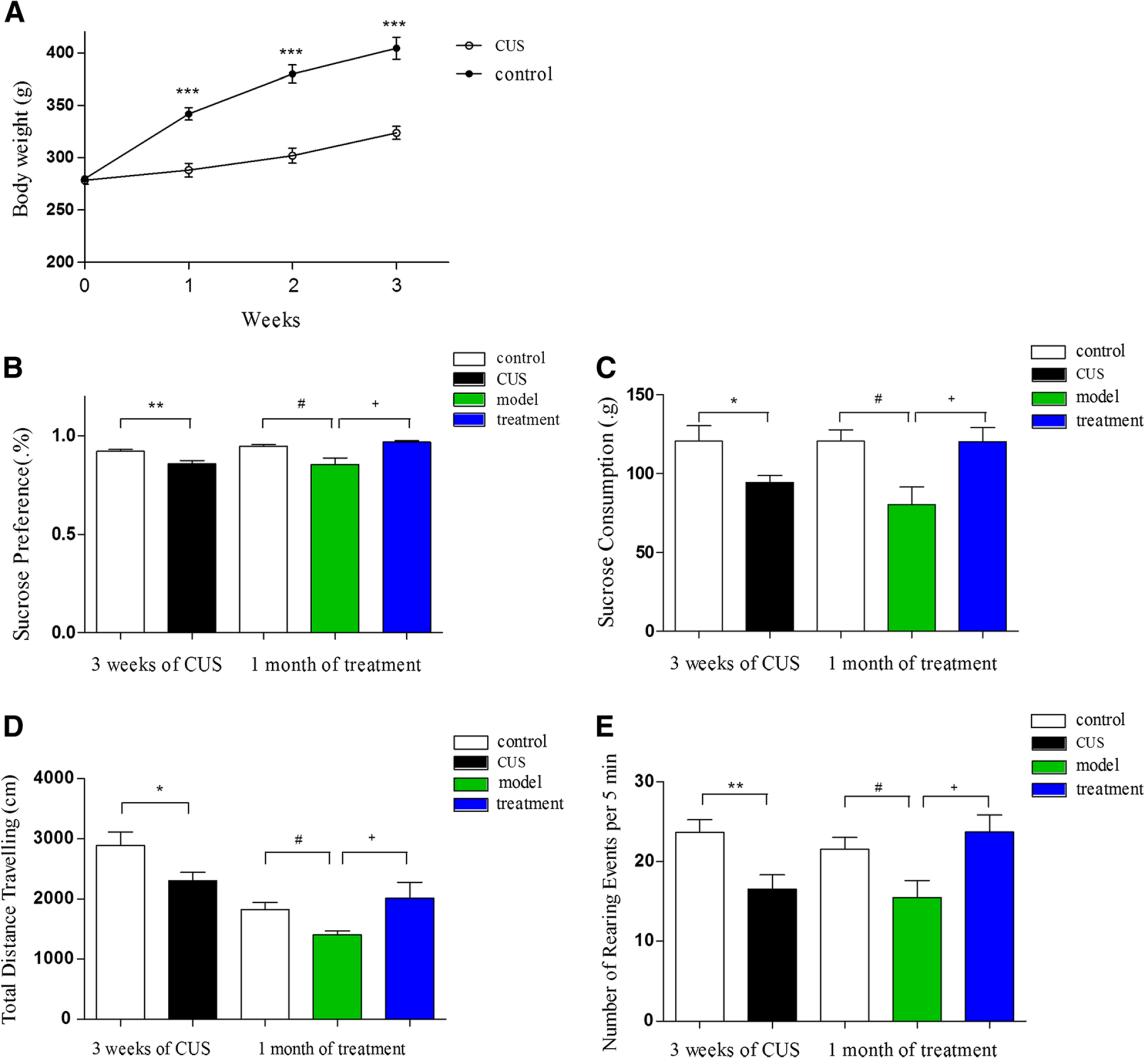
**Behavioral indicators changed during the CUS procedure and after curcumin treatment. (A)** Body weight changed during the 3 weeks of chronic unpredictable stress (CUS). ***P < 0.0001 shows the CUS group (n = 13) vs. control group (n = 14). **(B)** and **(C)** Sucrose preference and sucrose consumption after 3 weeks of CUS and after 1 month of treatment. *P < 0.05, **P < 0.01 shows the CUS group (n = 13) vs. control group (n = 14). ^#^*P* < 0.05 shows model group (n = 6) vs. control group (n = 14). ^+^*P* < 0.05 shows treatment group (n = 7) vs. model group (n = 6). **(D)** and **(E)** Open-field test indexes (total distance travelling and the number of rearing events) after 3 weeks of CUS and after 1 month of treatment. *P < 0.05, **P < 0.01 shows CUS group (n = 13) vs. control group (n = 14). ^#^*P* < 0.05 shows model group (n = 6) vs. control group (n = 14). ^+^*P* < 0.05 shows treatment group (n = 7) vs. model group (n = 6).

A repeated measurement ANOVA (time × group as factors) revealed a main effect of time (*F* = 80.42, *P* < 0.05), a main effect of group (*F* = 66.93, *P* < 0.05) and significant group by time interaction (*P* < 0.05). The control and CUS groups had similar body weights before the experiments (*P* > 0.05). After 3 weeks of chronic stress stimulation, the body weight gain in CUS group was significantly slower than that of the control group. These differences were found to be statistically significant (*P* < 0.05) (Figure 2A).

In the sucrose preference test, control and CUS groups showed comparable baseline sucrose preference and sucrose consumption (data not shown). However, after 3 weeks of chronic stress, both sucrose preference and sucrose consumption were significantly lower (5.5% and 6.7%) in the CUS group (Z = −2.706, *P* = 0.007. Figure 2B; Z = −2.228, *P* = 0.026. Figure 2C) than in the control group. One month later, the difference was further amplified to 8.7% and 18.6% (Z = −2.557, *P* = 0.011. Figure 2B; Z = −2.148, *P* = 0.032. Figure 2C). However, treatment with curcumin successfully reversed the effects of CUS, showing significantly more pronounced sucrose preference and sucrose consumption than in the model group (11.7% and 31.9%. Z = −2.429, *P* = 0.015. Figure 2B; Z = −2.286, *P* = 0.022. Figure 2C).

There was no difference between groups with respect to total activity during the 5 min open field test period (data not shown) at the baseline. However, after 3 weeks, CUS rats showed significantly less total distance travelling (23.0%) and significantly fewer rearing events (34.7%) than control rats (Z = −2.205, *P* = 0.027, Figure 2D; Z = −2.717, *P* = 0.007, Figure 2E). After one month, model rats also showed a significantly less total distance travelling (20.7%) and significantly fewer rearing events (23.8%) than control rats (Z = −2.062, *P* = 0.039. Figure 2D; Z = −2.072, *P* = 0.038, Figure 2E). Curcumin treatment in CUS rats was associated with significantly greater total distance travelling (20.7%) and significantly more rearing events (56.3%) than in the model group (Z = −2.143, *P* = 0.032. Figure 2D; Z = −2.295, *P* = 0.022. Figure 2E).

### Effects of CUS and curcumin on brain glucose metabolism

The differences in glucose metabolism in the coronal, sagittal, and horizontal sections among control rats, rats subjected to CUS, and CUS rats treated with curcumin (40 mg/kg) are shown in Table 2. Using Paxinos coordinates (ML, mediolateral; AP, anteroposterior; DV, dorsoventral), CUS induced strong activation of the right prim somatosens, right dorsolateral entorhinal cortex and basilar artery, and strong deactivation of the left mediodorsal thalami nuclear and cingulate cortex. One month later, model rats showed significantly more deactivation on both sides of the amygdalohippocampus than control rats. The administration of curcumin (40 mg/kg) induced strong activation of the left amygdalohippocampus and significant deactivation in the left primary auditory cortex.

**Table 2:** Parts of the brain showing changes in glucose metabolism

**Groups**	**Activation**	**Regions**	**Paxinos coordinate**^**a**^			**T-value**
			**ML**	**AP**	**DV**	
CUS vs. Control	Increase	Prim somatosens, upper lip(R)	6	−6	2	4.81
		Dorsolateral entorhinal cortex(R)	8	−8	−4	3.96
		Basilar artery	0	−12	−12	3.28
	Decrease	Mediodorsal thalami nu, central(L)	−1	−6	−3	−5.37
		Crus 2 of the ansiform lobule(L)	−5	−6	−12	−5.08
		Crus 2 of the ansiform lobule(R)	3	−4	−13	−4.04
		Caudate putamen(striatum) (R)	3	−7	0	−4.35
		Postsubiculum (L)	−3	−3	−7	−3.62
		Cingulate cortex, area 1	0	−2	4	−3.36
		Parietal cx,post area, rostral(R)	7	−2	−5	−3.23
Model vs. Control	Increase	Pyramidal decussation	0	−11	−16	2.7
	Decrease	Amygdalohip area, anterolat(R)	4	−9	−4	−2.86
		Amygdalohip area, anterolat(L)	−4	−9	−4	−2.31
Treatment vs. Model	Increase	Prim somatosens, forelimb(L)	−3	−1	1	3.5
		Amygdalohip area, anterolat(L)	−4	−9	−4	2.76
		Secondary somatosensory cx(R)	6	−6	−1	2.71
		4th cerebellar lobule	0	−1	−9	2
	Decrease	Primary auditory cortex(L)	−6	−4	−5	−3.34
		Spinal trigeminal nu, oral(R)	3	−9	−11	−2.23
		Pryamidal decussation	0	−11	−16	−2.01

^a^Paxinos coordinates point to the location of the voxel with the t-value ( *P* < 0.05 for the rest) in the designated brain areas. L, left; R, right.

**Figure 3:**
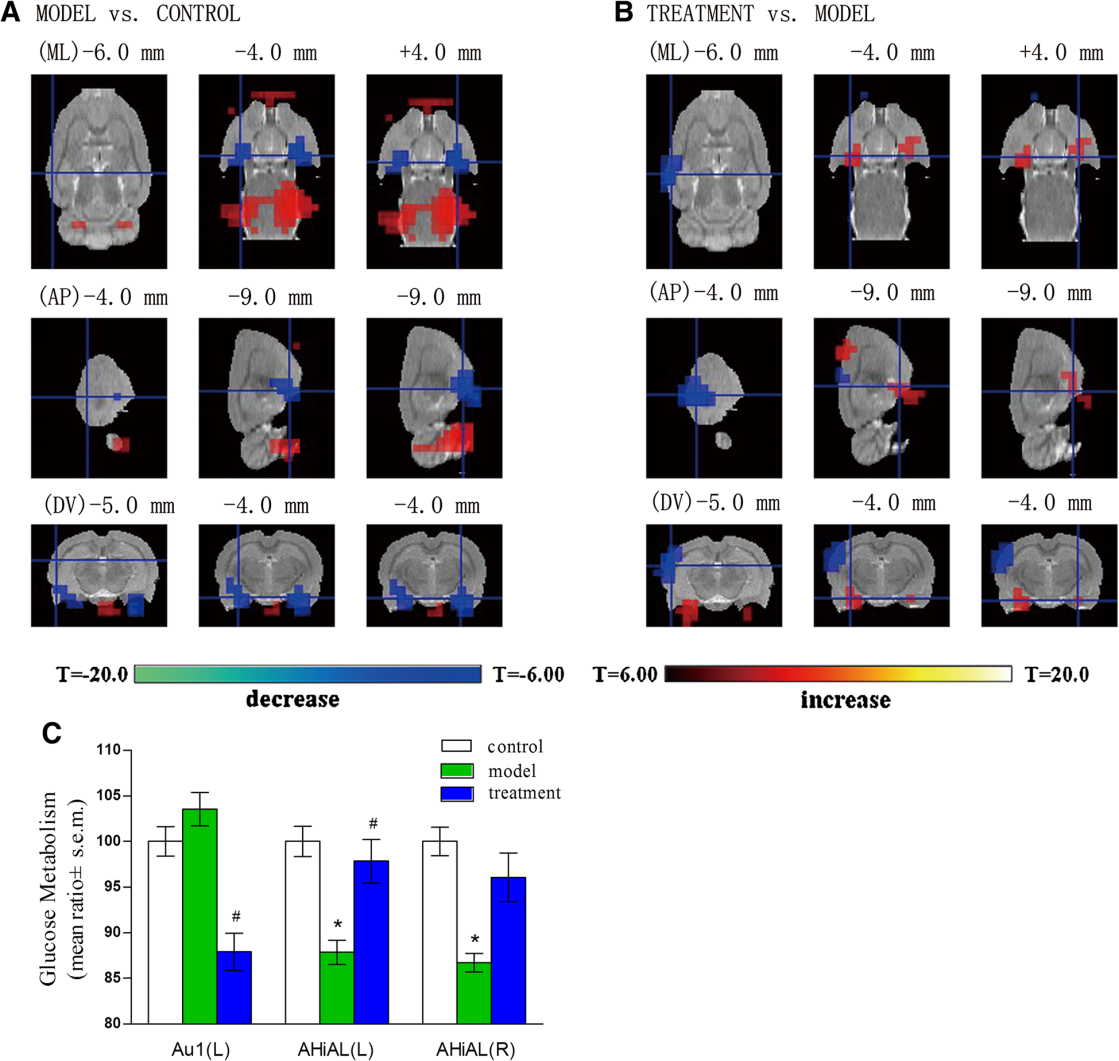
**Glucose metabolism in the brain changed during the CUS procedure and after curcumin treatment. (A)** Parts of the brain that showed significant changes in glucose metabolism induced by chronic unpredictable stress (CUS) relative to the control group. **(B)** Changes induced by CUS and the administration of curcumin compared with CUS group. **(C)** Changes in glucose metabolism in the brain. *Values differ significantly between model and control groups, # Values differ significantly between treatment and model groups, according to Mann–Whitney U (*P < 0.05 and ^#^*P* < 0.05). Au1, primary auditory cortex (ML: -6.0 mm, AP: -4.0 mm, DV: -5.0 mm); AHiAL (L), left amygdalohippocampal area, anterolateral part (ML: -4.0 mm, AP: -9.0 mm, DV: -4.0 mm); AHiAL(R), right amygdalohippocampal area, anterolateral part (ML: +4.0 mm, AP: -9.0 mm, DV: -4.0 mm).

Figure 3 summarizes these changes in several regions of interest in the three groups. Figure 3A shows the changes in glucose metabolism in coronal, sagittal, and horizontal sections in model rats and in the control group. The changes caused by curcumin comparing treatment group to model group as shown in Figure 3B. Figure 3C shows the administration of curcumin (40 mg/kg) inducing a strong activation of the left amygdalohippocampus (AHiAL, L). In contrast, significant deactivation was observed in the left primary auditory cortex (Au 1).

## Discussion

In this study, 3-week CUS significantly decreased body weight, sucrose preference, sucrose consumption, total distance travelling, and number of rearing events in rats. It also induced metabolic alterations in several parts of the brain, such as increased glucose metabolism in the right hemisphere. After one month of curcumin treatment, sucrose preference, sucrose consumption, total distance travelling, and number of rearing events returned to normal levels. These changes were associated with stronger deactivation of the left primary auditory cortex and activation of the amygdalohippocampal cortex than rats not given curcumin treatment. Overall, these data showed that curcumin could improve the behavioral and brain glucose metabolic abnormalities caused by CUS, suggesting that curcumin might have an antidepressive effect.

CUS was induced in male rats and their body weights were recorded for 3 weeks. The open field test is a common qualitative and quantitative measure of general locomotor activity and willingness to explore in rodents [24]. Open-field tests have shown a reduction in the total distance travelling and the number of rearing events in the CUS model, which can be considered indicative of the severity of depressive symptoms [20]. Consistent with a previous study [15], CUS rats here showed a decrease in sucrose consumption and sucrose preference, which is generally considered an indication of anhedonia [25]. The sucrose preference test is an objective behavioral indicator in rodent CUS model [26]. Here, this test showed that, after CUS, the rewarding value of sucrose solution was disrupted. Combined with the significant weight loss observed after CUS, these data indicate that the CUS model was effective.

The changes in the behavior in CUS rats were accompanied by alterations in glucose metabolism, specifically, increased glucose metabolism in the right prim somatosens, right dorsolateral entorhinal cortex, and basilar artery and decreased metabolism in the left mediodorsal thalami nuclear and cingulate cortex. In human beings, the right hemisphere is primarily responsible for dealing with negative emotions and the formation of pessimistic thinking. The left hemisphere is primarily responsible for processing pleasant experiences, and the relative decay can lead to anhedonia [27]. In neuroimaging studies, unipolar depressed patients show hypoactivity in the left hemisphere and hyperactivity in the right hemisphere [28,29]. The severity of depression has been shown to be positively correlated with right hemisphere hyperactivity [28]. In these ways, the abnormalities in glucose metabolism here observed in the CUS depression model was consistent with what has been observed in depressed patients, suggesting that this widely used animal model could also be used for further study of alterations in brain activity in depression.

One month of curcumin treatment significantly counteracted the decrease in four parameters, sucrose preference, sucrose consumption, total distance travelling, and number of rearing events. This suggests that curcumin might have a mild antidepressive effect. After one month of curcumin treatment at 40 mg/kg, the discrepancy in glucose metabolism between CUS and control groups was diminished. Nevertheless, several new parts of the brain showed significant differences, mainly gathered at amygdalohippocampus and left primary auditory cortex. It might be due to an extension of the CUS effect, primarily non-responsive parts of the brain start reacting to stress as time goes on.

Both sides of the amygdalohippocampus showed significantly more deactivation in the model rats than control rats, while the treatment of curcumin played a role in resistance to such changes in the present study. In previous studies, patients with major depressive disorders showed abnormal activation in the amygdala when presented with emotional stimuli [30]. These data indicated that the amygdala is involved in the etiopathology of depression and that curcumin might improve emotional processing. The serotonin hypothesis states that serotonin might be an important explanation for the present observed results. It has been reported that curcumin strongly increased the serotonin levels and inhibited monoamine oxidase in mice [5]. One report has suggested that acute serotonin reuptake blockage may affect human amygdala reactivity [31]. In this way, the activation of glucose metabolism in the amygdalohippocampus might contribute to an increase in serotonin caused by curcumin. The amygdalohippocampus, a part of the cortical-amygdal loop, is involved in emotional processing. Previous studies have shown that the amygdalohippocampal area receives dense serotonergic input [24,25]. The serotonin hypothesis of depression suggests that levels of serotonin metabolites are low in depressed individuals and that levels of central serotonin are low in the brains of suicide victims [25]. The CUS model also suggests a decrease in serotonin metabolites in the frontal cortex, striatum, and hippocampus [26]. Confusingly, previous studies have demonstrated that the resting cerebral blood flow and glucose metabolism are abnormally elevated in the amygdala in patients with major depressive disorder but not in patients with unipolar depression [32]. These data seem to conflict with the present results, but three major factors might explain this contradiction. There are profound differences between different species difference, such as human beings and rodents. There is also a dearth of effective research that illustrates whether emotional processing is consistent across these different species. Another possibility is that, in the present study, the amygdalohippocampus, the caudal third of the amygdala which abuts the hippocampal formation, but not the whole amygdala, showed significant deactivation in model rats. It is possible that different parts of the amygdala have diverse responses to CUS and curcumin, but further research is needed to confirm these conclusions. It should be noted that the amygdala does not show over-activation in patients with unipolar depression [32], which indicates that abnormally elevation of amygdala may be associated with the manic phase. Previous studies have shown that stimulating the amygdala caused the animal to become more active and to attempt to escape the stimulation, indicating defensive behaviors [33]. Further investigation is needed to address these questions.

Conversely, the auditory cortex showed a significant deactivation after curcumin treatment. The auditory cortex is closely related to the amygdalohippocampus and is also rich in serotonergic fibers. The excitability of the auditory cortex is assumed to be under serotonergic control. Decreases and increases in serotonin function are associated with increased and reduced cortical excitability, respectively [34]. Consistent with this suggestion, the present results showed that curcumin may act as a monoamine oxidase inhibitor can increase the serotonin levels, which cause the deactivation of the auditory cortex. Curcumin, which has multiple bioactivities, also functions as a potent inhibitor of neuronal cell death in response to oxidative stress in auditory neurons [35]. However, the exact mechanism by which curcumin affects cerebral glucose metabolism requires further investigation.

Previous functional neuroimaging studies using ^133^Xe blood flow, single photon emission computed tomography (SPECT), and PET have consistently demonstrated decreased regional cerebral blood flow (rCBF) or metabolism in the frontal lobe [36], temporal lobe [37], or anterior cingulate gyrus [38] of depressed patients. Our results also demonstrated that curcumin can alleviate CUS induced stress through improving cerebral glucose metabolism. Although it is still lack of clinical test, the antidepressant effect of curcumin has been proved by availability of vast preclinical evidence [6], implying curcumin can alleviate the symptoms of depression by various mechanisms, such as inhibiting monoamine oxidase enzyme, modulating the level of various neurotransmitters, and promoting hippocampal neurogenesis [6]. Thus, based on these studies, it will be helpful for curcumin or related agents translational development in the future.

However, there are some limitations in the present study. First, micro PET technology itself has certain limitations. This may bias the statistical analysis when relatively large parts of the brain are involved, so the present study only measured changes in glucose metabolism in small parts of the brain rather than in the hippocampus or prefrontal cortex. Second, the present study involved a small sample size. Finally, the biggest limitation of the current study is not clearly specified on hypothesis by which mechanism curcumin affects cerebral glucose metabolism. According to the present results, our future research will aim to test the hypothesis of curcumin against depression by inhibiting monoamine oxidase in a large sample.

## Conclusions

In summary, CUS induced weight loss and depression or anxiety behaviors in rats. These changes were accompanied by metabolic alterations in several parts of the brain regions, specifically increased glucose metabolism in the right hemisphere. Curcumin treatment reversed these depression and anxiety behaviors and induced strong deactivation of the left primary auditory cortex and activation of amygdalohippocampal cortex. These data suggest that curcumin may act as an antidepressive agent, possibly by reversing the abnormal brain glucose metabolism that occurs in depressed organisms.

## Competing interests

The authors declare that they have no competing interests.

## Authors’ contributions

ZL conceived and designed the study; ZL, JL, JHL and LGS participated in the experiment; ZL and LGS drafted the manuscript; HH and YRL helped to draft the manuscript; CTZ and WJT participated in performed the statistical analysis; AMB reviewed and edited the manuscript; LX conceived of the study, and participated in its design and coordination and helped to draft the manuscript. All authors read and approved the final manuscript.

## Pre-publication history

The pre-publication history for this paper can be accessed here:

http://www.biomedcentral.com/1472-6882/13/202/prepub
